# Comparison of patient preferences and responsiveness among common patient-reported outcome measures for hand/wrist injuries or disorders

**DOI:** 10.1186/s10195-022-00681-4

**Published:** 2023-01-09

**Authors:** Phongniwath Chanthana, Pichitchai Atthakomol, Worapaka Manosroi, Tinakon Wongpakaran, Jirachart Kraisarin, Kanit Sananpanich

**Affiliations:** 1 Department of Orthopaedics, Faculty of Medicine, Chiang Mai University, Chiang Mai, Thailand; 2 Musculoskeletal Science and Translational Research Center, Chiang Mai University, Chiang Mai, Thailand; 3 Clinical Epidemiology and Clinical Statistic Center, Faculty of Medicine, Chiang Mai University, Chiang Mai, Thailand; 4 Division of Endocrinology, Department of Internal Medicine, Faculty of Medicine, Chiang Mai University, Chiang Mai, Thailand; 5 Department of Psychiatry, Faculty of Medicine, Chiang Mai University, Chiang Mai, Thailand

**Keywords:** Patient-reported outcome measures, Patient preference, Responsiveness, Hand/wrist, Injuries, Disorders

## Abstract

**Background:**

Patient-reported outcome measures (PROMs) were developed to examine patients’ perceptions of functional health. Most studies compare the responsiveness of each type of questionnaire. However, reports of patient preferences among PROMs commonly used with patients with hand/wrist injuries or disorders are limited. This study aimed to compare patient preferences, factors associated with those preferences and responsiveness among the Disability of the Arm, Shoulder, and Hand (DASH), Michigan Hand Outcomes Questionnaire (MHQ), Patient-Rated Wrist/Hand Evaluation (PRWHE) and EQ-5D in patients with hand/wrist injuries or disorders.

**Material and methods:**

This retrospective cohort study collected data on 183 patients with hand/wrist injuries or diseases who had visited a hand/wrist outpatient clinic or were hospitalized for surgery between 2017 and 2020. Patients had to be at least 18 years old and able to complete the four questionnaires included in the study. The four PROMs (DASH, MHQ, PRWHE and EQ-5D) were administered to the patients prior to treatment. After completing the questionnaires, patients were asked to answer two open-ended questions regarding their preferences. Multinomial logistic regression was used to identify factors related to patient preferences. Results are presented as the relative risk ratio (RRR). The standardized response mean (SRM) was used to evaluate questionnaire responsiveness.

**Results:**

Of the 183 patients, most preferred the PRWHE questionnaire (*n* = 74, 41%), with the main reasons cited being “specific to injuries/diseases and reflects hand/wrist function (*n* = 23, 31%)” and “easy to complete (*n* = 22, 30%).” Sex was found to be associated with patient preference after adjusting for demographic data and reasons for choosing a PROM as confounders (RRR = 0.46, *P* value = 0.049). The PRWHE had the highest SRM, followed by DASH (0.92 and 0.88, respectively).

**Conclusions:**

The PRWHE is the most preferred by patients and is the most responsive questionnaire. It is recommended for use in clinical practice in situations where a clinician would like to use only one PROM for evaluating patients with various types of hand/wrist problems.

*Level of evidence*: Prognostic III.

## Introduction

An adjunct to physical examination and radiography after therapy, patient-reported outcome measures (PROMs) were developed to examine patients' perceptions regarding functional health, which represents the true success of treatment [1, 2]. Currently, PROMs are used not only in the assessment of treatment outcomes in clinical research, but are also increasingly being employed as part of a healthcare approach that includes the measurement of outcomes from the perspective of the patient [3]. Commonly used tools for assessing hand and upper extremity disability status include the Disability of the Arm, Shoulder, and Hand (DASH), the Michigan Hand Outcomes Questionnaire (MHQ) and the Patient-Rated Wrist/Hand Evaluation (PRWHE), all of which provide good psychometric properties [4–6]. The EQ-5D questionnaire is widely used to assess general health and to evaluate changes in clinical outcomes of patients with a distal radius fracture [7].

In the past, many studies have focused on a comparison of the responsiveness of different types of questionnaires to aid in identifying the best outcome measurements for detecting clinical change and to determine the smallest difference in the score of an outcome instrument that patients perceive as important in cases of a specific type of injury or disorder [8–16]. In actual clinical practice, hand surgeons often treat a variety of hand/wrist injuries or disorders. In those situations, the clinician may use more than one PROM depending on the nature of the injury/disease. However, interpretation of the results depends on the specific scoring system of each PROM [17–21]. Identifying the best patient-reported outcome measure that can be used with many different types of injuries or disorders would provide benefit in terms of generalizability and ease of interpretation of the results. The concept of patient preferences is an important factor that could potentially improve patient–doctor collaboration for decision making in many aspects of a patient’s care [3]. However, there have been only a limited number of studies focusing on the comparison of patient preferences among PROMs.

The decision regarding which PROM to use to assess a patient’s hand/wrist symptoms or function depends on the judgement of the clinician and may not reflect the patient’s symptoms or the severity of the injury. A better understanding of patient preferences in terms of performance, ease of completion and precision of evaluation of hand/wrist function can assist in the selection of the most appropriate questionnaire [22, 23].

Identifying a specific PROM for a patient with hand/wrist injuries or disorders that provides both a high ability to detect clinical change over time and which is preferred by patients for describing their symptoms and the severity of their injury in actual clinical practice could potentially provide significant benefits, especially when a clinician would like to use only one PROM to evaluate patients with various types of hand/wrist problems. The aim of this study was to compare patient preferences, factors associated with preferences and responsiveness among four commonly used PROMs (PRWHE, MHQ, DASH, EQ-5D).

## Material and methods

This retrospective study was conducted at a university hospital and was approved by the institutional ethics committee (ethical number: ORT-2564–08550). We collected data on 183 patients with hand/wrist injuries or diseases who had visited a hand/wrist outpatient clinic or were admitted for surgery between 2017 and 2020. Inclusion criteria were aged at least 18 years old and able to complete the four PROMs. Demographic data, including age, sex, dominant hand, injured hand, diagnosis, education level and employment status, were recorded. The Thai versions of the PRWHE, MHQ, DASH and EQ-5D were given to all the patients prior to treatment [24–27]. After completing the four questionnaires, patients were asked to respond to two open-ended questions. (1) Which questionnaire best addressed your symptoms or the severity of your injury during this clinical experience? (2) Please explain why you selected that particular questionnaire. The name of the questionnaire that the participants chose was recorded. Written informed consent was obtained from all patients. This study adhered to the Strengthening the Reporting of Observational Studies in Epidemiology (STROBE) Statement [28].

The PRWHE questionnaire comprises 15 items grouped into three subscales: pain, specific activities, and usual activities. Response options range from 0 to 10, with lower scores indicating less pain or disability. The total score for the PRWHE ranges from 0 to 100, with lower scores indicating better functional hand use [21].

The MHQ questionnaire consists of 37 items to assess six subscales: overall hand function, activities of daily living, pain, work performance, aesthetics, and patient satisfaction with hand function. Four of these six sections inquire separately about how the right and left hands are impacted. Scores are normalized and summed for a total of between 0 and 100, with 100 representing excellent perceived hand function [19].

The DASH questionnaire contains 30 items in five subscales: common activities; self-care activities; pain symptoms; other symptoms including numbness, joint stiffness, weakness, and sleep problems and psychological effects; and optional sports and work modules, each with five response options (1–5). Lower scores indicate better functional hand use. Total scores range from 0 to 100, with 0 representing no difficulty in the performance of daily tasks [17, 18].

The EQ-5D questionnaire is a two-part outcome measurement which is extensively used to evaluate health status [29, 30]. The first part consists of five subscales: mobility, self-care, usual activities, pain/discomfort, and anxiety/depression. Each subscale has five levels of severity, ranging from no problems to extreme problems. The second part uses numeric scales to evaluate general health condition, with scores ranging from 0 to 100, where higher scores indicate better health.

All four PROMs had previously been translated into Thai and cross-culturally adapted following standard guidelines, and were shown to have adequate internal consistency in all subscales as well as good construct validity and reliability with Thai patients [24–27, 31–33].

The Thai PRWHE, MHQ and DASH were scored manually, following the original scoring algorithms [17, 19, 21], while the scores of the Thai EQ-5D were analyzed using the EQ-5D-5L Crosswalk Index Value Calculator, which is available for ten countries: Denmark, France, Germany, Japan, the Netherlands, Spain, Thailand, the UK, the US and Zimbabwe (https://euroqol.org/eq-5d-instruments/eq-5d-5l-about/valuation-standard-value-sets/crosswalk-index-value-calculator/).

### Statistical analysis

For demographic data, categorical variables are reported as frequencies and percentages. Continuous variables are reported as means and standard deviations. Statistical significance was set at *P* < 0.05.

Patient questionnaire preferences and reasons for selecting a particular questionnaire are reported as percentages of participants.

Univariable and multivariable analysis (multinomial logistic regression) were performed to identify factors related to patient preference. Potential factors were chosen from the demographic data and from reasons given by patients for selecting a particular questionnaire. Factors associated with patient preferences are reported as the relative risk ratio (RRR) with the 95% confidence interval (95% CI).

Responsiveness is defined as the ability of a measurement to detect clinically significant changes over time [34]. Responsiveness of the four PROMs was evaluated by comparing the scores at baseline and at follow-up periods using the standardized response mean (SRM) and effect size (ES). SRM is the observed mean change divided by the standard deviation of the observed change, while ES is the observed mean change divided by the standard deviation of the baseline scores. SRM is the preferred value for comparing paired data measurements at different time points for the same patient. SRM and ES values of 0.8, 0.5, and 0.2 were considered to be large, moderate, and small, respectively [15, 35].

Floor or ceiling effects were considered to be present if more than 15% of patients reported the lowest or highest possible scores [36]. In these patients, the responsiveness is reduced because the changes cannot be evaluated [34]. The data were analyzed using Stata Statistical software 15 (Stata Corp, LP, College Station, TX, USA).

## Results

The mean age of the 183 patients involved in this study was 45 years (SD 17), with females predominant (*n* = 122, 67%). Most were right-hand dominant (*n* = 167, 91%). The ratio of injured hand between right and left was comparable (*n* = 84, 46% vs. *n* = 76, 41%). The three most frequent diagnoses were tendon entrapment (*n* = 47, 26%), hand/wrist fracture (*n* = 40, 22%) and nerve entrapment (*n* = 28, 15%). The majority of the patients had a secondary/high school education or equivalent (*n* = 65, 35%) followed by a bachelor’s degree (*n* = 51, 28%). Most patients were employed (*n* = 107, 59%) (Table 1).

**Table 1:** Characteristics of the patients (*n* = 183)

Characteristics	Value
Age (years), mean (SD)	45 (SD 17)
Female, *n* (%)	122 (67)
Right-hand dominant, *n* (%)	167 (91)
Injured hand, *n* (%)	
Right	84 (46)
Left	76 (41)
Both	23 (13)
Diagnosis	
Tendon entrapment	47 (26)
Hand/wrist fracture	40 (22)
Nerve entrapment	28 (15)
Tumor	17 (9)
Finger deformity from tendon injury	11 (6)
Joint arthritis	10 (5)
Joint dislocation	7 (4)
Ligament injury	6 (3)
Joint stiffness	6 (3)
Hand infection	3 (2)
Others	8 (4)
Education level, *n* (%)	
Elementary school	34 (19)
Secondary/high school or equivalent	65 (35)
Diploma	17 (9)
Bachelor’s degree	51 (28)
Higher than a bachelor’s degree	16 (9)
Employment status *n* (%)	
Employed	107 (59)
Unemployed	46 (25)
Retired	30 (16)

Most of the patients in our study preferred the PRWHE questionnaire (*n* = 74, 41%), citing as the main reasons that the PRWHE is “specific to injuries/diseases and reflects hand/wrist function” (*n* = 23, 31%) and that it is “easy to complete” (*n* = 22, 30%). The second most preferred questionnaire was the MHQ (*n* = 61, 33%). The main reasons patients preferred the MHQ were that it “provides high detail” (*n* = 22, 36%) and that it is “easy to complete” (*n* = 18, 30%) (Table 2).

**Table 2:** Patients’ PROM preferences and reasons for those preferences (*n* = 183)

Reason	PRWHE, *n* (%)	MHQ, *n* (%)	DASH, *n* (%)	EQ-5D, *n* (%)
Provides high detail	9 (12)	22 (36)	6 (32)	0 (0)
Easy to complete	22 (30)	18 (30)	9 (47)	13 (45)
Specific to injuries/diseases and reflects hand/wrist function	23 (31)	8 (13)	0 (0)	2 (7)
Concern for overall health status	0 (0)	0 (0)	0 (0)	7 (24)
Covers both sides of hand/wrist	0 (0)	2 (3)	0 (0)	0 (0)
Others	11 (15)	2 (3)	3 (16)	2 (7)
No reason	9 (12)	9 (15)	1 (5)	5 (17)
Total	74 (41)	61 (33)	19 (10)	29 (16)

Evaluation of factors associated with patient preferences found that female patients were more likely to select the PRWHE to address their symptoms or severity of injury than the MHQ (RRR = 0.46, *P* value = 0.049), adjusting for age ≥ 65 years, diagnosis, education level of at least a bachelor’s degree, employment status and reason for patient preference as confounders. Age ≥ 65 years, diagnosis, education level of at least a bachelor’s degree, being employed and reason for patient preference were not statistically significantly associated with the patient’s choice of preferred questionnaire (*P* value > 0.05) (Table 3).

**Table 3:** Factors related to patient PROM preference (*n* = 183)

PROM	Factor	Univariable analysis			Multivariable analysis		
		RRR	95% CI	*P* value	RRR	95% CI	*P *value
MHQ	Age ≥ 65 years	1.07	0.36–3.14	0.903	0.82	0.23–2.88	0.757
	Female	0.43	0.21–0.90	0.025*	0.46	0.21–0.99	0.049*
	Diagnosis	1.05	0.96–1.16	0.283	1.03	0.93–1.14	0.589
	Education level at least a bachelor’s degree	1.56	0.77–3.13	0.214	1.26	0.60–2.66	0.539
	Employed	1.51	0.95–2.38	0.079	1.49	0.87–2.55	0.142
	Reason for choice	0.88	0.76–1.03	0.114	0.89	0.76–1.05	0.164
DASH	Age ≥ 65 years	0.97	0.19–5.00	0.972	0.98	0.15–6.47	0.980
	Female	0.36	0.13–1.02	0.054	0.34	0.12–1.00	0.051
	Diagnosis	0.95	0.81–1.13	0.593	0.93	0.80–1.09	0.398
	Education level at least a bachelor’s degree	1.43	0.51–3.99	0.500	1.28	0.43–3.76	0.659
	Employed	1.26	0.64–2.48	0.513	1.20	0.55–2.61	0.653
	Reason for choice	0.83	0.64–1.07	0.151	0.83	0.64–1.08	0.166
EQ5D	Age ≥ 65 years	1.32	0.37–4.77	0.672	0.81	0.19–3.52	0.779
	Female	0.84	0.32–2.23	0.732	1.00	0.35–2.85	0.995
	Diagnosis	1.08	0.96–1.21	0.190	1.09	0.96–1.24	0.172
	Education level at least a bachelor’s degree	0.62	0.23–1.66	0.344	0.48	0.17–1.38	0.174
	Employed	1.43	0.81–2.53	0.221	1.76	0.88–3.55	0.111
	Reason for choice	1.06	0.89–1.26	0.517	1.08	0.91–1.29	0.386

* Statistically significant at P-value <0.05

PRWHE (base outcome) is PRWHE was used as the baseline questionnaire. Other questionnaire (MHQ, DASH, EQ5D) was compared to PRWHE in each aspect.

More than half the patients in the study received surgical treatment (57%). Fifty-one patients were followed up, at which time they completed each of the questionnaires, including the evaluation of the perceived responsiveness. The mean time to follow-up was 2 months. No floor or ceiling effects were found in the total scores of any of the four PROMs. The results show that the PRWHE was rated as the most responsive questionnaire, with the highest SRM (0.92) and ES (1.0), followed by the DASH questionnaire, with SRM = 0.88 and ES = 0.76 (Table 4).

**Table 4:** Standardized response mean (SRM) and effect size (ES) of each questionnaire (*n* = 51)

Questionnaire	Baseline mean (SD)	Mean follow-up (SD)	Mean difference (SD)	SRM	ES
PRWHE	56.77 (SD 20.45)	36.25 (SD 26.17)	20.52 (SD 22.39)	0.92	1.00
MHQ	50.36 (SD 19.92)	65.03 (SD 21.55)	14.66 (SD 17.40)	0.84	0.74
DASH	54.38 (SD 17.01)	41.39 (SD 17.71)	12.99 (SD 14.77)	0.88	0.76
EQ5D	0.51 (SD 0.23)	0.68 (SD 0.23)	0.16 (SD 0.23)	0.73	0.70

Mean time to follow-up = 56.25 days (SD 27.77)

Treatment: surgical treatment—29 patients (57%); conservative treatment—22 patients (43%)

*DASH* Disabilities of the Arm, Shoulder and Hand, *MHQ* Michigan Hand Questionnaire, *ES* effect size, *SD* standard deviation, *SRM* standardized response mean

## Discussion

This study found that patients with hand/wrist injuries or disorders preferred the PRWHE and felt it was the most responsive questionnaire among the four commonly used PROMs (PRWHE, MHQ, DASH, EQ-5D). This study compares both patient preferences and responsiveness among commonly used PROMs, while previous studies have focused primarily on comparing the responsiveness of various PROMs. This suggests that the PRWHE is appropriate for use by clinicians to evaluate clinical outcomes in a general hand/wrist clinic.

In terms of patient preference, the two top questionnaires were the PRWHE and MHQ, both of which are specific to patients with hand/wrist problems. Each questionnaire has its own special advantages. The PRWHE has only 15 items which are specific to pain and function, while the MHQ addresses 37 items in 6 subscales and requires specifying the right or left hand, which increases the time taken to complete the MHQ. DASH is a more general questionnaire which focuses on symptoms and the functional status of the upper extremities with 30 items, and EQ-5D has only 5 items, which are used to evaluate general health. A previous study of 59 patients with hand fractures which described patient PROM preferences in terms of ease of completion and measurement of ability to use the hand reported that the PRWHE was easiest to complete and that the MHQ best reflected ability to use the hand [1]. In the present study, open-ended questions were used to identify the preferred questionnaire, rather than having participants select among a list of specific reasons, which potentially could have influenced patients to select a specific questionnaire. In the current study, we assumed that patients would prefer a questionnaire which is specific to their hand/wrist problem (the PRWHE and MHQ). Among the questionnaires specific to their hand/wrist problem, patients tended to choose the questionnaire which was easiest to complete (the PRWHE). The current study found that sex was associated with the choice of preferred questionnaire: females generally preferred the PRWHE, while most males preferred the MHQ. Previous publications have also reported a correlation between sex and decision-making for various issues in both humans and animals [37, 38]. However, we were unable to identify reasons why females tended to prefer the PRWHE.

The questionnaire which participants found to be most responsive in our study was the PRWHE, followed by the DASH and MHQ, in that order. The PRWHE, MHQ and DASH have all been previously reported to be commonly used PROMs for the hand/wrist region. Most studies have compared responsiveness between either the MHQ and DASH [8, 9, 11, 16] or between PRWHE/PRWE and DASH/QuickDASH [10, 13, 21]. We decided to include EQ-5D in this study because there is supporting evidence that EQ-5D provides acceptable to good responsiveness among patients with a distal radius fracture [7]. Previous comparisons of responsiveness between the MHQ and DASH have reported a slightly higher SRM with the MHQ than with DASH in patients with hand injuries at the 3-month follow-up [8, 11]. In patients with carpal tunnel syndrome, wrist pain and tumors, however, at the 3-month follow-up, the SRM of DASH was higher than that of the MHQ [9]. The PRWHE/PRWE has been reported to provide a higher SRM than DASH in patients with hand or wrist problems [10, 13, 21]. A study comparing responsiveness among the PRWHE, MHQ and DASH in patients with a hand fracture reported that the MHQ had the highest SRM, followed by DASH and the PRWHE at the 3 months follow-up [1]. It is important to note that these differences in responsiveness among the PRWHE, MHQ and DASH were based on a variety of factors, e.g., different diagnoses, treatments and follow-up periods.

The population in the present study would be expected to focus on pain and hand function, which suggests why the PRWHE demonstrated a higher responsiveness compared to the MHQ and EQ-5D, which include evaluations of aspects of aesthetics and mobility, respectively.

In our cohort, the three most common hand/wrist problems, in descending order, were tendon entrapment (trigger digit, de Quervain’s disease), hand/wrist fracture and nerve entrapment. In previous studies, tendon entrapment and nerve entrapment have been reported to be commonly found in stable populations in Greece and the United Kingdom [39, 40]. However, in our cohort there were no patients with Dupuytren’s disease, a common hand disorder in Western countries which is relatively rare in Asians [39, 41–44]. Most of the patients with hand/wrist injuries or disorders in this study were female, which is similar to other studies [39, 40]. Based on patient characteristics, we determined that our cohort was a good representative sample of the general population. Although many subscales have been recommended for the evaluation of PROMs, our results clearly show that in evaluating pain and function, the PRWHE is able to detect major concerns and clinically significant changes over time in patients who have common hand/wrist problems.

There were several strengths of our study. First, we focused on patients’ preferences as well as on their perception of the responsiveness of PROMs commonly used with patients who have hand/wrist problems, unlike prior studies, which have focused primarily on responsiveness. Second, the number of PROMs evaluated and the size of the population in our cohort were relatively high.

A limitation of this study is that only one-third of the patients in the study evaluated the responsiveness of all four common PROMs. Eighty-nine patients were lost to follow-up after receiving the treatment, and 43 patients did not complete all four common PROMs when they came to follow-up. However, evaluating responsiveness with 51 patients is roughly comparable to previous studies [8, 10, 11, 15, 16, 21].

Among four common PROMs (PRWHE, MHQ, DASH, EQ-5D) for the evaluation of patients with hand/wrist injuries or disorders, the PRWHE is the most preferred by patients and the most responsive questionnaire. The PRWHE is recommended for use in situations where clinicians would like to use only one PROM for evaluating patients with various types of hand/wrist problems.

## Data Availability

The datasets used and/or analyzed during the current study are available from the corresponding author on reasonable request.

## Abbreviations

CI: Confidence interval
DASH: Disability of the Arm, Shoulder, and Hand
ES: Effect size
MHQ: Michigan Hand Outcomes Questionnaire
PROMs: Patient-reported outcome measures
PRWHE: Patient-Rated Wrist/Hand Evaluation
RRR: Relative risk ratio
SD: Standard deviation
SRM: Standardized response mean
STROBE: Strengthening the Reporting of Observational Studies in Epidemiology
